# License Plate Recognition Algorithm for Passenger Cars in Chinese Residential Areas

**DOI:** 10.3390/s120608355

**Published:** 2012-06-15

**Authors:** Lisheng Jin, Huacai Xian, Jing Bie, Yuqin Sun, Haijing Hou, Qingning Niu

**Affiliations:** 1 Transportation and Traffic College, Jilin University, Nanling Campus, 5988 RenMin Street, Changchun 130022, China; E-Mail: xianhc11@mails.jlu.edu.cn; 2 Faculty of Engineering Technology, University of Twente, P.O. Box 217, 7500 AE Enschede, The Netherlands; E-Mail: J.Bie@ctw.utwente.nl

**Keywords:** license plate recognition, image processing, character localization, character segmentation, character

## Abstract

This paper presents a solution for the license plate recognition problem in residential community administrations in China. License plate images are pre-processed through gradation, middle value filters and edge detection. In the license plate localization module the number of edge points, the length of license plate area and the number of each line of edge points are used for localization. In the recognition module, the paper applies a statistical character method combined with a structure character method to obtain the characters. In addition, more models and template library for the characters which have less difference between each other are built. A character classifier is designed and a fuzzy recognition method is proposed based on the fuzzy decision-making method. Experiments show that the recognition accuracy rate is up to 92%.

## 1. Introduction

License plate recognition is an important issue in the field of intelligent transportation systems. It utilizes computer vision and pattern recognition technologies [1,2]. A successful license plate recognition system relies on the robust performance of both its hardware and software components. The main functions of the hardware components are vehicle detection, vehicle image acquisition and data transmission. The software components take care of vehicle image preprocessing, license plate localization, character segmentation and character recognition.

For license plate localization, widely used methods and techniques include edge statistics, mathematical morphology [3], connected domain analysis [4], regional segmentation [5], color model transformation [6,7], fuzzy set theory [8] and statistical classification [9]. The localization rate varies from 80% to 97%.

According to the license plate characteristics, projection, histogram and classifier methods are adopted for segmentation in the character segmentation module [10–13]. In addition, the specific algorithms and segmentation rates are improved. In the character recognition module, the methods of template matching [14], neural network [15,16], support vector machine, cascade classifier, Markov net and Bayes net also have been used for license plate recognition [17–20].

In recent years, some western countries such as U.S., Singapore, Japan, Canada, Germany, Italy, U.K., and France have developed license plate recognition systems and successfully applied them to their own traffic management. License plate recognition algorithms need to operate fast enough to meet the requirements of intelligent transportation system [20–22]. Nevertheless, with the rapid development in digital image processing technology, the detection and identification of license plate takes less than 50 ms, so 20 frames of video images [21] can be processed in one second. In the study described in [23], the system was implemented on an embedded DSP platform and the system processes a video stream in real-time. This system consisted of detection and character recognition modules. The method used for detecting license plates was AdaBoost. Detected license plates were segmented into individual characters by using a region-based approach. In order to improve the embedded platform processing speed, a Kalman tracker was inserted into the system and was used to forecast the position of the license plate in the next frame image. The real-time processing was the biggest advantage in this system. In addition, it didn't require any additional sensor inputs (e.g., infrared sensors), in addition to a video stream.

From the study of [24], the license plate recognition system was advanced by adopting a cascade framework. A method of fast identification algorithms was developed by using the characteristics of license plate characters. The system, which was composed of three cascading modules for plate detection, character segmentation and post processing, could recognize the license plates at over 38 frames per second and the recognition rate was higher than 90%.

In China, the license plate consists of several types of characters, including Chinese characters, Latin letters and numbers. Due to the differences between Chinese characters and other font, the license plate recognition systems in Western countries are not completely suitable for China. Therefore, it is necessary to develop a license plate recognition system specifically for China. Since the 1990s, many Chinese companies and scholars have been devoting time to this area of research. A recognition rate of 90% has been achieved but only for good visibility conditions.

In this paper, we focus on the requirements of the civil vehicle management system and the characteristics of civil vehicle license plate, a license plate identification algorithm and a license plate recognition system for use in the community were developed. In the license plate recognition system, image processing and pattern recognition technology were adopted.

## 2. Image Pre-Processing

### 2.1. Image Acquisition

**Figure 1.**
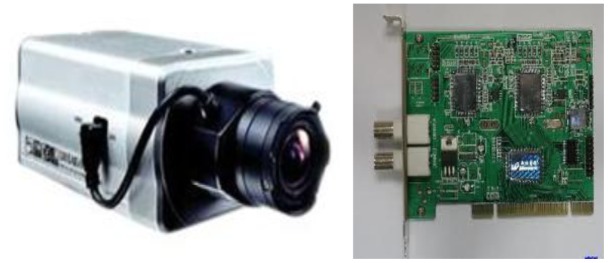
The camera of YT-3501-T and V221 acquisition card.

Radar sensors detect vehicles entering the residential area and send out requests for image acquisition. A camera installed at the entry gate will then take photos of the vehicle. In this study, the YT-3501-T color camera (Figure 1) is adopted. The image acquisition system is composed of color camera, automatic aperture lens and a V221 acquisition card (Figure 1). Horizontal distance from camera to the license plate is 2,300 mm and vertical distance is 1,450 mm. Under these distance conditions, the license plate picture accounts for 1/5 to 1/3 in area of the whole vehicle picture. If images are acquired at night, a metal halide lamp installed at a height of 1,600 mm is used as auxiliary lighting equipment. In addition, it should also be noted that, the camera must avoid the lights of the oncoming vehicles. The picture resolution can set manually; 640 × 480 is adopted in this study.

### 2.2. Image De-Noising

When the license plate recognition systems are applied in outdoor areas, they are affected by weather and lighting conditions, as well as the complex backgrounds. This may bring noise to the acquired images. Noise is inevitable, but most of the noise can be eliminated with a smoothing filter. Through the de-noising procedure, subtle fractures can be linked and tiny abrupt parts can be softened. The principles that appear to be the most relevant to the image de-noising are preventing the original image edge from being destroyed, retaining the image outline and lines as much as possible, keeping the continuity of the image and increasing the contrast between regions of interest and not of interest. In this study, the standard median filter which has important features such as time saving, high precision and good performance is used.

**Figure 2.**
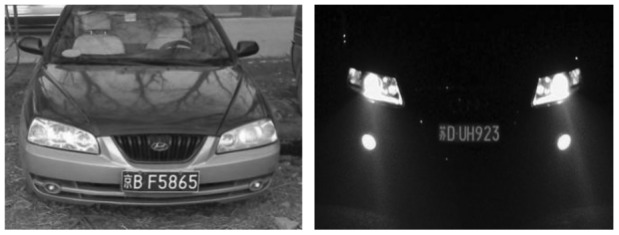
Original images.

**Figure 3.**
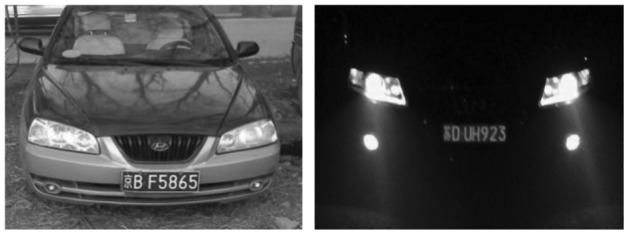
Results of median filter.

The center pixel in the scan window is to be de-noised. The first step is to sort all the pixel values in the scan window and find the mid-value, then change the mid-value into the standard median of the sorted sequence. Figures 2 and 3 show the experimental results. Through this method, information of image edges is saved as much as possible and the contrasts between regions of interest and not of interest are increased.

### 2.3. Edge Detection

The edge of images reflects the information such as boundary of the area, brightness discontinuity, texture changes and surface orientation, *etc.*, so it is also the important basis of regional segmentation in interested areas. In addition, image edge detection also can remove irrelevant details and noise.

**Figure 4.**
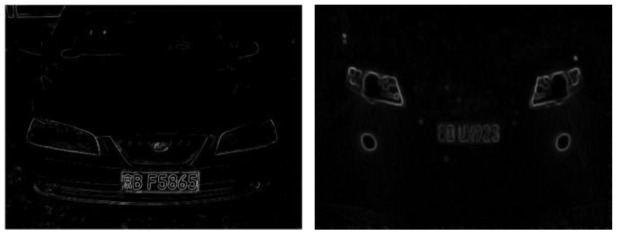
Edge detection.

In China, license plate contains seven characters, including Chinese characters, Latin letters and numbers. Because of the structure of Chinese characters is complicated, the number of strokes is more and texture change is comparatively obvious. From Figure 4, it is noticed that compared with other areas, the gray area of the license plate changes more frequently and the edge information is richer. So the edge detection can be used to extract license plate area, separate prominent targets and background. This paper adopts method of Sobel edge detection [25,26]. In Figure 4, the detection effect is shown.

### 2.4. Binarization

In the procedure of digital image processing, the step of binarization can reduce the invalid information, highlight the outline information of a target area and improve the follow-up processing speed. According to the threshold value, binarization methods can be divided into the methods of global threshold value, local threshold value and dynamic threshold value.

During the license plate recognition procedure, the global threshold value method provides better computation efficiency if the vehicle images are obtained under uniform illumination and without noise, but in most cases, the environment around the license plate is complex and the illumination is uneven, so the performance of a single binary threshold is poor. The deficiency of the local threshold value method is that it sometimes destroys the consistency between neighboring blocks, and the binary image may appear deformed or fuzzy. When the headlights are turned on at night, the illumination around the license plate is uneven. Therefore methods using global or local threshold values do not produce ideal results, whereas the Gaussian Laplace operator can achieve better results by using a dynamic binary.

**Figure 5.**
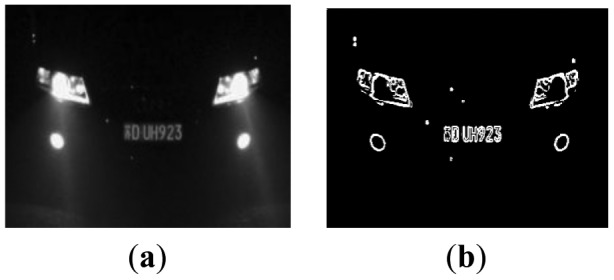
Binarisation of night vehicles. (**a**) Gray image; (**b**) Results of binarisation.

**Figure 6.**
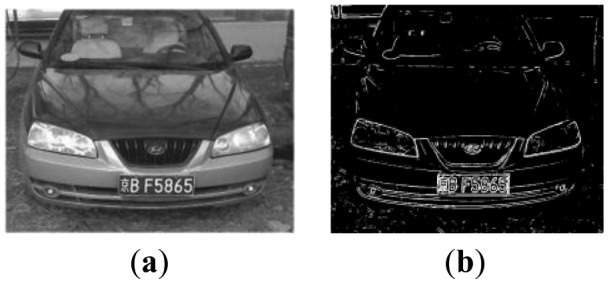
Binarisation of day vehicle. (**a**) Gray image; (**b**) Results of binarisation.

In this study the Gaussian Laplace operator is combined with an iterative method. When illumination is even, the iterative method is adopted; when illumination is uneven, the Gaussian Laplace operator is adopted. Firstly, the gray level histogram in normal illumination is analyzed and an optimal threshold H is calculated. Secondly, the total value of the accumulative gray image in each vehicle image is compared with H. If this value is greater than H, the second method is used; if it is less than or equal to H, the first method is applied. The results are shown in Figures 5 and 6.

## 3. License Plate Localization

The starting point of the license plate location is to judge the license plate through the features of the car license plate area. Available license plate features include five aspects: (1) that the geometrical features of the license plate, that is the height, width and their proportions, are within the confines; (2) the form feature is that the license plate is in a rectangular frame and characters are arranged according to certain rules in the rectangular frame with intervals; (3) the gray distribution of car license plate area feature is that the horizontal lines through the license plate have a gray distribution of continuous peaks and troughs; (4) the horizontal or vertical projection characteristics of car license plate area present a continuous peaks and troughs distribution and (5), the spectrum processes the image by row or column DTF transformation and its diagram contains the location information of the license plate.

According to the rules set in 2007 by the People's Republic norm GA36 2007 mobile license plate standard in China, the basic characters of a vehicle license plate are as follows:

1. Color features. China has the following color placements for the license plate background and characters: blue background with white letters, and yellow-black, black-white, white background with red or black letters, *etc*. The color of background and character form a sharp contrast, and the license plate color is not consistent with the body color. In the surroundings, there is a low chance of finding the same color schemes, so the color can be used as a feature for license plate location.
2. Outline Size characteristics. The license plate size of small cars in China meets the standard X3–X7, with each character being 5 mm-wide and 90 mm-high. The space is 10 mm between Chinese characters and letters, while it is 12 mm between characters. It can be obtained according to the prior knowledge that the license plate location in the original image changes within a certain scope.
3. Character features. In vehicle images, areas around the license plate are, compared to other areas, rich in edge points and texture, and rectangular with a fixed aspect ratio [27,28]. These unique features are adopted to distinguish the license plate from its background. Accordingly a license plate recognition method is developed based on fusion of significant features. This method improves the accuracy and adaptability of license plate localization.

### 3.1. Approximate Localization Based on Texture Feature

**Figure 7.**
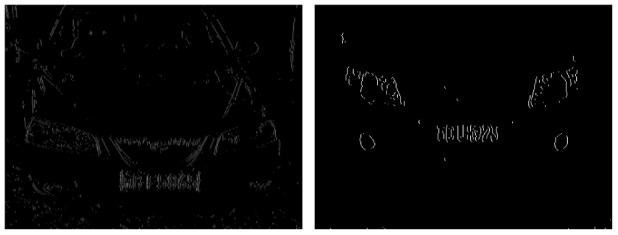
Images of edge points.

As seen in Figure 7, for areas around the license plate, the number of edge points in each row is typically between 120 and 250, the ratio between the number of edge points and the length of license plate area is from 3.9 to 13, the number of jump points is from 13 to 40.

The first step of approximate localization is to detect the column range of the license plate. The whole image is scanned. Then the number of edge points, the number of jump points and the ratio which is between the number of edge points and the length of license plate area are calculated. If the column range meets the numerical interval changes range, this line and the starting point are marked, then repeating this process.

The second step is to detect the row range. Through previous steps, the row range of license plate is obtained. In order to confirm the row range, we set the max and min values of the row range, the number of edge points in each row, the interval between starting point and end point.

**Figure 8.**
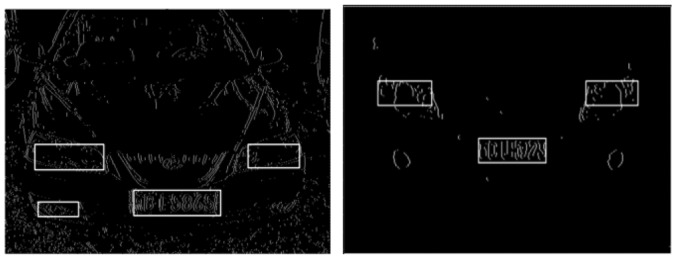
Approximate localization results.

License plate boundary is determined in the second step. As is shown in the edge points image, the starting point and end point of each row are concentrated and only a few lines are deviating. A statistical analysis of the distribution of these two points is made. The starting point and end point which appears most frequently are chosen as the boundaries of the license plate. Through the above steps, a few appropriate areas are found and the approximate localization is completed. Approximate localization results are shown in Figure 8.

### 3.2. Accurate Localization Based on Aspect Ratio

**Figure 9.**
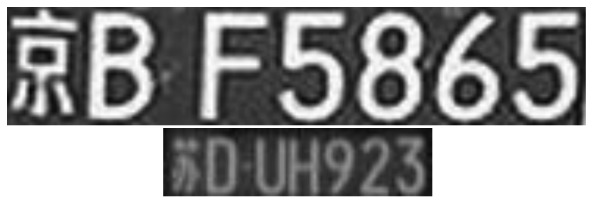
Accurate localization.

In order to eliminate pseudo license plate areas, the aspect ratio of each candidate region is calculated. Accurate localization is shown in Figure 9.

## 4. Skew Correction of License Plate and Character Segmentation

### 4.1. Skew Correction of License Plate

Because the camera and the license plate are not located at the same height (also because of road slope and vibration of the vehicles), the images of license plate exhibit a certain degree of skew. This study develops a skew correction method with high speed of operation, simple structure and high accuracy. The procedure of this method is described below.

First, the left half of the image is scanned and the average height of white pixel is calculated, denoted as “leftaver”. Then, the right half is scanned and “rightaver” is calculated. The slope is then determined by the follow formula.

(1) slope=(leftaver−rightaver)/(nWidth/2)

### 4.2. Character Segmentation

**Figure 10.**
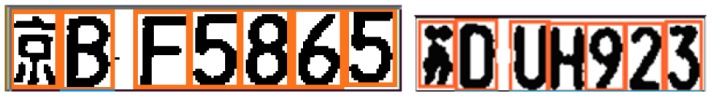
Character segmentation.

The method of row-column scan is chosen to segment characters. Firstly, the line scan method is used to scan the binary image and lower-upper bounds are located. Secondly, the column scan method is chosen to scan binary image and the left-right bounds are located. Based on these, each character can be accurately segmented. Experimental results show that this method can even handle license plate images with fuzzy, adhering, or fractured characters with high efficiency. The primary steps of character segmentation are as follows:

- Fluctuation boundary of characters is identified. The area of the license plate is scanned from top to bottom and the position of the first scanned white pixel point is marked as the top boundary. Then, scanning from bottom to top, the position of the first scanned white pixel point is marked as the bottom boundary.
- Border around the characters is confirmed. Columns are scanned gradually from left to right and position of the first scanned white pixel point is marked as the left boundary. *white_num*=1 is recorded as the number of pixels and then continuously, *white*_*num*++. Because every character in the license plate has its ratio of high to width, the letter R represents the ratio of high to width. *iHeight/white_num* is the ratio of high to width which is scanned by computer. If *iHeight/white_num* > 2 × *R* and no white pixel is in whole column, character segmentation is not end. The work is completed until no white pixels are found in a whole column and *iHeight/white_num* = *R*, at this time, right boundary of the first character is confirmed.
- If during the course of scanning, from *white_num* = 1 to *iHeight/white_num* = *R*, no white pixels is found in a whole column, the character images must be joined, so the characters are divided mandatorily. A rectangular box is used to divide the characters. In order to avoid overlap, the rectangular box expands a pixel.
- The position information of division character is putted in the structure *rect* and the structure is inserted in behind of the linked list *charrect*1, and an assigned position is set. The next character's segmentation is begun.
- Steps (b) to (d) are repeated. If *i* ==(*nwidth*-1), the last letter's right boundary is confirmed, the linked list *charrect*1 is used to lay out the seven characters of the license plate.
- Every character in the linked list *charrect*1 is scanned again and the height-width accurate location is checked. Finally the linked list *charrect*1 is assigned to *charrect*2. The segmentation result of some license plate characters is shown in Figure 10.

### 4.3. Character Normalization

The sizes of characters from different images are varied, which hinders feature extraction and recognition, therefore character normalization and thinning processing are essential. Character normalization is a procedure of arrangement of various characters into a uniform size according to a prepared template.

**Figure 11.**
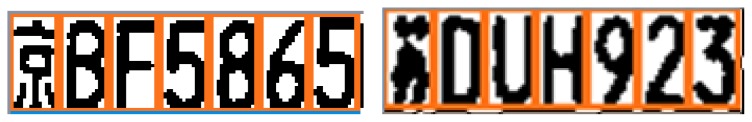
Character normalization.

During the course of character normalization, the height and width of segmentation character are compared with those of a standard character. According to matrix zoom, zoom factors Rx and Ry are ascertained. The top boundary and left boundary remain unchanged. According to standard character, the bottom boundary and right boundary are determined. The last step is defining a new structure is to to lay out a rectangular box. The size of a normalized character is 16 × 32. Results of character normalization are shown in Figure 11.

### 4.4. Character Thinning Processing

The characters after normalization have the same size, however, what makes the recognition more tedious and difficult is that their fonts don't have an uniform diameter and the width of their strokes are more than single pixels. Thinning processing cuts the points around the contour layer by layer based on a certain processing algorithm, which refines the character strokes to the width of a single character and removes the redundant information, and then we obtain the character skeleton including image features and basic structures. Principles we need to follow include:

1. The continuity of character strokes should be kept so as to prevent the strokes fracture.
2. The character skeleton should approach the centerline of the strokes as close as possible and the width should be a single pixel.
3. The original geometric characteristics and topological structure should be preserved, and the endpoints of lines should not be deleted.
4. No serious deformation is allowed after thinning.

**Figure 12.**
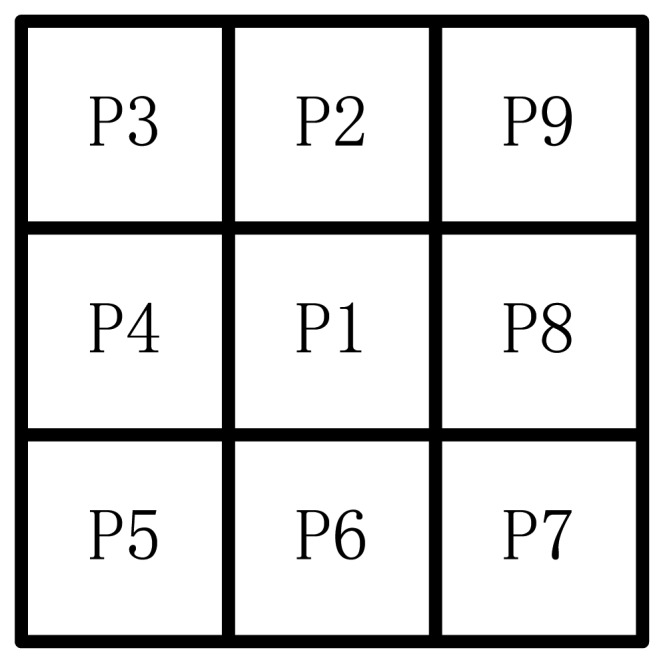
The marked point and neighbor points.

This study adopts the following methods to calculate the structure of the target area. The pixels of the backdrop area are marked as 0, and the pixels of the target area and boundary area are marked 1. At least one point's pixel is 0 in any boundary point joint areas. The marked point is supposed as *P*1, and *Pi*(*i* = 2,3,…9) are the neighbor points. The marked point and neighbor points are shown in Figure 12.

The area of 3 × 3 in one image has nine points: *P*1, *P*2,…*P*9, *P*1 is in the center of the area. If *P*1 = 1 and satisfaction follows four conditions, *P*1 is deleted.

- 2 ≤*NZ*(*P*1) ≤6
- *Z*0(*P*1)=1
- *P*2 × *P*4 × *P*8 = 0 or *Z*0(*P*1) ≠1
- *P*2 × *P*4 × *P6* = 0 or *Z*0(*P*4) ≠1

**Figure 13.**
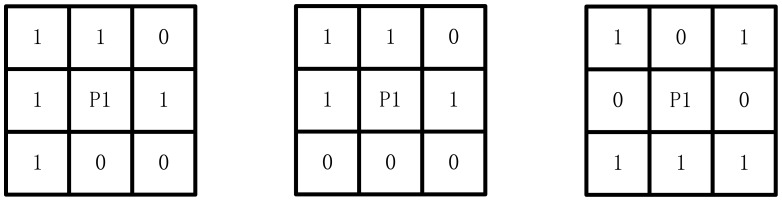
Conditions of P1 preservation.

*NZ*(*P*1) is the number of non-zero points in *P*1, *P*2,…*P*9, Z0(*P*1) is the scale of pixel diversification. The condition (1) deletes some inner things and the neighbor points with only one pixel value. The condition (2) confines the processing area to a single pixel. In this condition structure rupture is avoided. The conditions (3) and (4) delete the possibility that *P*1 is a boundary point. Figure 13 shows some *P*1 that are preserved under any condition.

**Figure 14.**
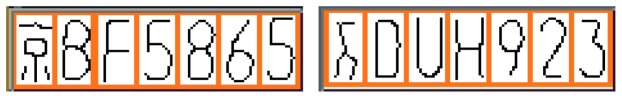
Thinning processing.

All the boundary points are examined gradually. If the points are in accordance with the above conditions, we mark them as 0. If the point is marked 1 at the start, these points are preserved. The process above is repeated no more points are deleted in images. The remaining points form the structure of the character area. The results of the thinning process are shown in Figure 14.

## 5. Character Recognition

Character recognition means that the related characteristic information of pending recognized characters is extracted first, analyzed and classified. Secondly, template matching with the algorithm for recognition of plate characters is applied. Finally, a template which is the most similar to the identification character is found and the recognition of characters is realized. The main steps in character recognition include feature extraction and classifier of character, character model library construction and character reorganization. The chosen character features and feature extraction are two key factors in the character recognition system.

### 5.1. Feature Extraction

Character feature extraction is to choose a set of parameters as eigenvectors which are the most representative of the character feature, namely the best characteristic attribute measurement of samples that determines the ability of an identification system. At present, the widely used license plate character features include two categories: structural characteristics and statistical characteristics.

**Figure 15.**
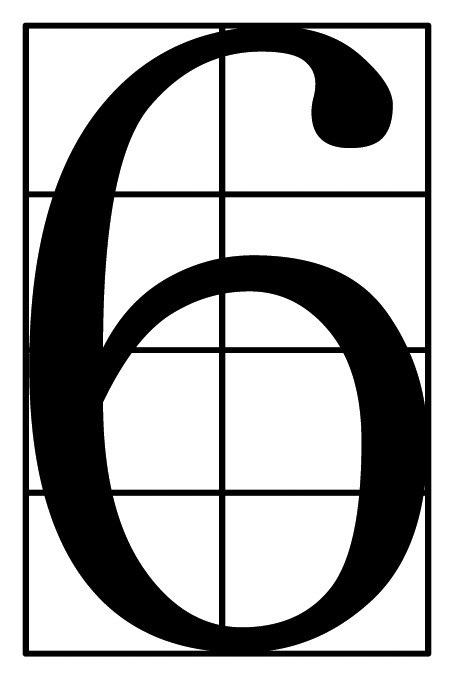
Eight characteristics of a number.

There is a trade-off between using structural characteristics and using statistical characteristics for character recognition. Structural characteristics are better suited for distinguishing similar characters but are difficult to extract and unstable. Using statistical characteristics contributes to a better robustness in the recognition algorithm, but its discriminatory power is poor for similar characters. Thus in this paper, the structural characteristics and the statistical characteristics are combined to extract features. Firstly, the grid features are extracted for rough classification; secondly, the internal structure characteristics are extracted to distinguish the similar characters. Compared with the single feature extraction method, this method can greatly improve recognition rates and shorten the recognition time. The specific procedures are as follows:

First of all, the grid features are extracted. Taking the character “6” as an example, it is divided into 8 parts, and this segmentation situation is shown in Figure 15. The number of black pixels in each part is taken as eight characteristics.

**Figure 16.**
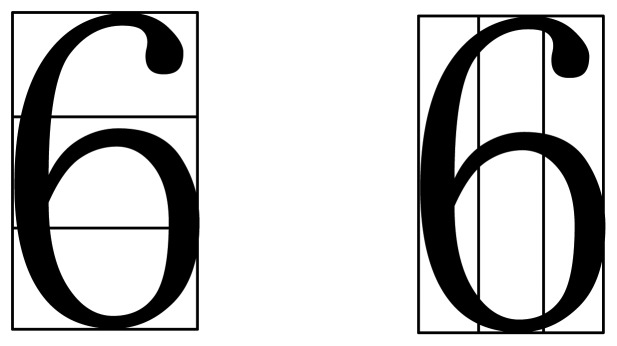
Four characteristics of a number.

Second, the internal structure characteristics are extracted. From previous steps, we can see that the grid features of some characters are similar, such as “B” and “ 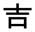
”, “ 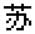
” and “8”ôwhich are difficult to distinguish. Therefore, it is necessary to extract the internal structure characteristics to distinguish between similar characters. Two horizontal and vertical mid-columns must be found, between which four lines should be drawn. The black pixel points through the four lines are calculated as 4 characteristics, respectively, as shown in Figure 16. Finally, all black pixel points should be counted as one of 13 total characteristics.

### 5.2. Character Classifier Design

The standard form of civil license plates in China is *X_1_X_2_* × *X_3_X_4_X_5_X_6_X_7_*. Here *X_1_* is a Chinese character representing the provincial level division; *X_2_* is an upper case Latin letter; *X_3_*−*X_7_* are letters or Arabic numerals, among which there are at most two letters except “I” “O” “D”.

**Figure 17.**
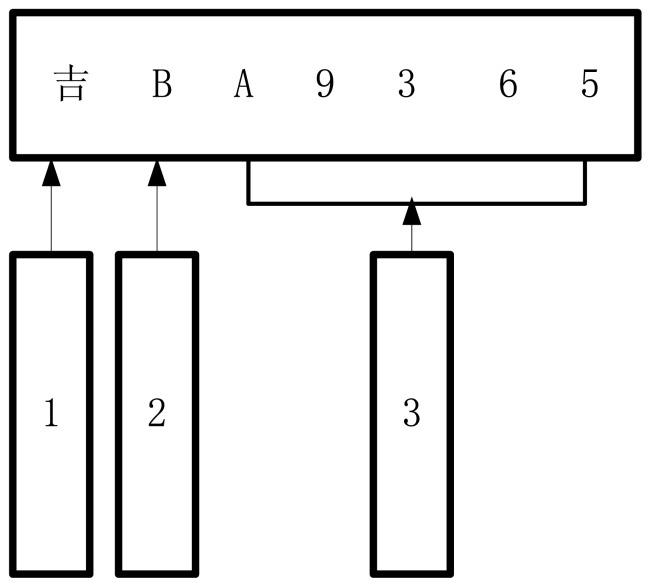
Three types of classifier.

In order to improve the speed and rate of recognition, three character classifiers are designed. They are a Chinese character classifier, Latin letter classifier, and Numbers-Letters classifier. The characters of the license plate from left to right should be recognized and the corresponding classifier according to their serial number are chosen. The three kinds of classifier are shown in Figure 17.

### 5.3. Building Character Model Library

The mean and standard deviation characteristics are selected to describe the character. N characteristics and H sample of character M are expressed as:

(2) $$M(p)=\left[m_{p1},m_{p2}\ldots m_{pn}\right],p=1,2\ldots H$$

(3) $$m_{j}=\frac{\overset{H}{\underset{i=1}{\text{∑}}}m_{ij}}{H},j=1,2,\ldots n$$

(4) $$m_{j}^{\prime }=\sqrt{\frac{1}{H}\sum_{p=1}^{H}{\left(m_{p}-m_{p1}\right)}^{2}},j=1,2\ldots n$$

where, *M*(*P*) is the characteristic value of each sample; *P* is serial number of sample; *n* is the serial number of a characteristic; *m_j_* and $m_{j}^{\prime }$ are the mean and standard deviation of the characteristics, respectively.

### 5.4. Fuzzy Decision of Character Recognition

**Figure 18.**
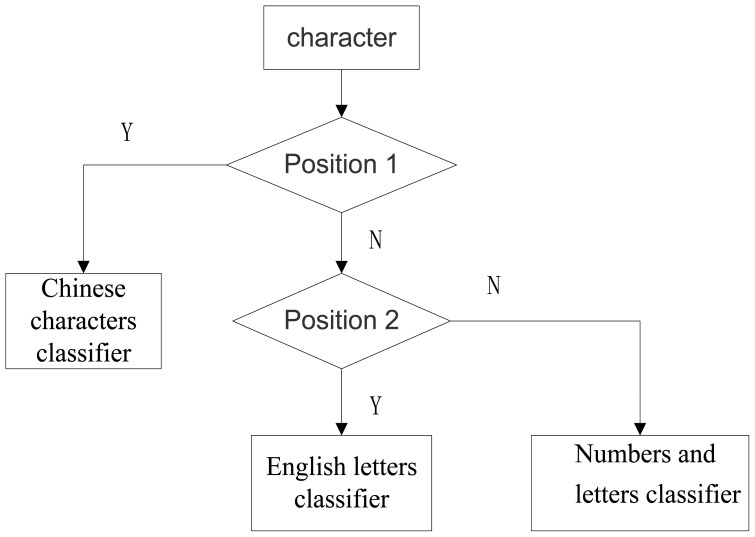
Position classification flow diagram.

The 13 dimensional feature vector of the character of i which is input in the sequence of i should be calculated. In addition, the *S*[*i*] of the 13d feature vector also should be calculated. *S*[*i*] is a distance weighted value of every character in template set. *S*[*i*] means degree of feature difference between the characteristics of the characters and template characters. The recognition result is the minimum of *S*[*i*]:

(5) $$S\left[i\right]=\sum_{k=1}^{13}\alpha (k)\times \left\{\text{fea}[i][k]-\text{temp}[j][k]\right\}$$

where *α*(*k*) is metric; *fea*[*i*][*k*] is the characteristics of characters that is to be identified; *temp*[*j*][*k*] is the characteristics of template characters. The specific procedure is as follows:

Firstly, according to the position, each character is categorized by the different classifiers, shown in Figure 18. Secondly, the characters that are to be identified with template characters are compared and their matching degree is calculated by the following equation:

(6) $$S\left[i\right]\left[k\right]=\{\begin{array}{cc} \left|\text{fea}\left[i\right]\left[k\right]-\text{temp}\left[j\right]\left[k\right]\right|, & k=1,2\\ 2\times \left|\text{fea}\left[i\right]\left[k\right]-\text{temp}\left[j\right]\left[k\right]\right|, & k=3,4,5,6\\ 0.5\times \left|\text{fea}\left[i\right]\left[k\right]-\text{temp}\left[j\right]\left[k\right]\right|, & k=7,8\\ \left|\text{fea}\left[i\right]\left[k\right]-\text{temp}\left[j\right]\left[k\right]\right|, & k=9,10,11\\ 2\times \left|\text{fea}\left[i\right]\left[k\right]-\text{temp}\left[j\right]\left[k\right]\right|, & k=12,13 \end{array}$$

**Figure 19.**
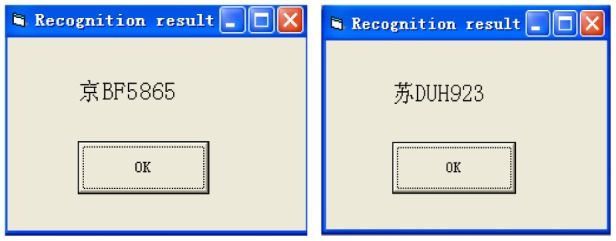
Recognition results.

Thirdly, when matching degree reaches a minimum value, the corresponding template character is the recognition result, which is shown in Figure 19.

## 6. License Plate Recognition Experiment

**Figure 20.**
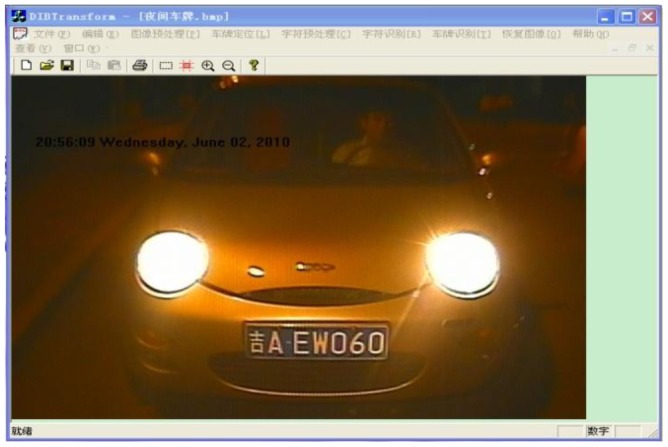
Interface image.

**Figure 21.**
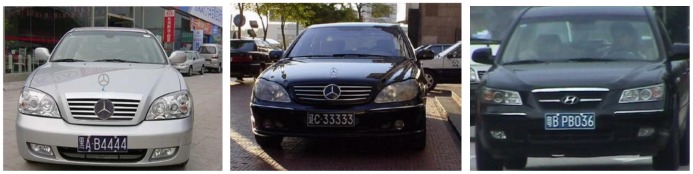

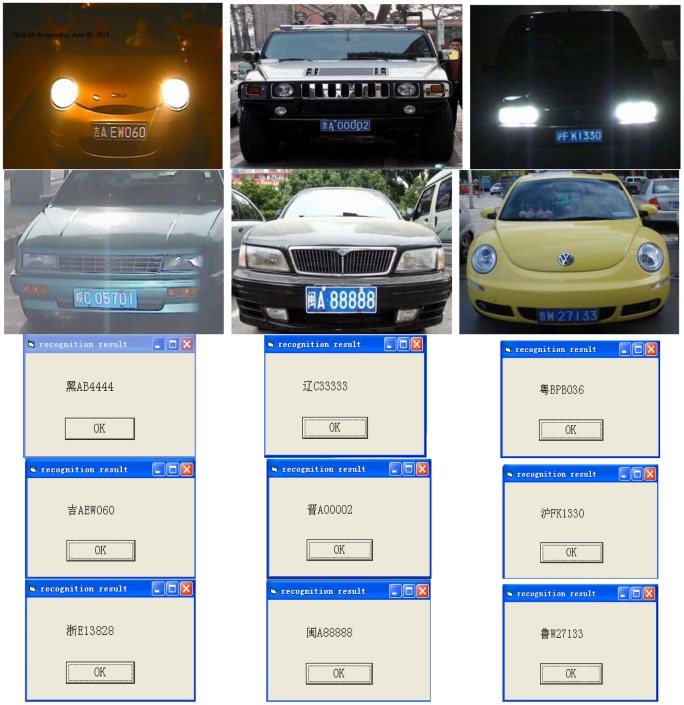
The original images and reorganization results.

The license recognition algorithm has been programmed using the VC++ 6.0 platform. The interface is shown in Figure 20. The algorithm has been tested under natural scenes in Chang Chun (China). The test pictures are taken by a CCD camera. Each picture contains only one license plate and has a resolution of 640 × 480. The test results are shown in Figure 21.

A total of 300 vehicles images obtained from community entrance gates have been tested. The recognition algorithm shows a high accuracy rate of 92%. We can conclude that the license plate recognition algorithm proposed here is capable of identifying passenger car license plates against complex backgrounds.

## 7. Conclusions

In this paper, application software is designed for the recognition of civil vehicle license plates. License plate images were pre-processed and the plate locations were extracted first. Then, we corrected the skew of license plates and separated the plate characters individually by segmentation. Finally, according to the features of Chinese letters, we applied template matching with the use of an algorithm for recognition of plate characters. This system is designed for the identification of Chinese license plates and was tested over a large number of images. Finally through license plate recognition experiments, it was proven that the system designed in this study for Chinese license plate reorganization performed with better than 92% recognition rates.

Some tasks are still needed in the next step of this paper. Due to the effects of character noise, such as the fracture and adhesion of the license plates' key parts, it is necessary to make further selection of multi-features to represent characters. The location and segmentation of more license plates against complex backgrounds also need further study.
